# Supplementary material for: Dual Insecticidal Effects of Adenanthera pavonina Kunitz-Type Inhibitor on Plodia interpunctella is Mediated by Digestive Enzymes Inhibition and Chitin-Binding Properties
Source: Molecules. 2019 Nov 28;24(23):4344. doi: 10.3390/molecules24234344 (PMC6930628; doi:10.3390/molecules24234344)
Supplement: Supplementary file 1 [file molecules-24-04344-s001.pdf]

Table S1. *In silico* interactions between ApKTI/N-acetylglucosamine; ApKTI/trypsin and ApKTI/chymotrypsin.

**ApKTI (gi: 124152/ gi: 124153)**				**N-acetylglucosamine**			
Arg	66	NH2	3.2	GlcNAc	1	O1	HB
Arg	64	NH1	3.5	GlcNAc	2	O7	HB
Arg	64	NH2	3.5	GlcNAc	3	O6	HB
Glu	77	OE2	3.6	GlcNAc	5	O7	HB
Thr	75	OG1	3.1	GlcNAc	6	O7	HB
Gln	112	OE1	3.0	GlcNAc	6	O4	HB
**ApKTI (gi: 124152/ gi: 124153)**				**Trypsin (gi: 157137123)**			
**Residues**	**Positions**	**Atom Names**	**Distances (Å)**	**Residues**	**Positions**	**Atom Names**	**Interactions**
Gly	115	O	3.6	Arg	55	NH2	HB
Arg	138	NH2	3.4	Thr	24	OG1	HB
Thr	75	OG1	3.5	Arg	55	NE	HB
Glu	77	OE1	2.7	Thr	53	OG1	HB
Arg	64	NH1	3.1	Gly	50	O	HB
Ala	54	N	3.6	Glu	77	OE2	HB
Ser	60	OG	2.7	Ala	52	O	HB
**ApKTI (gi: 124152/ gi: 124153)**				**Chymotrypsin (gi: 1336053)**			
Glu	80	OE2	3.0	Ser	53	O	HB
Asn	91	ND2	3.6	Pro	83	O	HB
Lys	95	NZ	3.3	Trp	85	O	HB
Glu	109	OE1	3.0	Asn	84	O	HB
Glu	109	OE2	3.6	Thr	91	OG1	HB
Arg	138	NH1	3.0	Thr	232	OG1	HB
Arg	138	NH2	3.0	Thr	232	OG1	HB
Pro	62	O	3.2	Ala	236	O	HB
Arg	64	NH2	3.5	Leu	238	O	HB
Ser	107	O	3.5	Asn	86	ND2	HB

Å: Ångström; **gi**: accession number in NCBI; **HB:** Hydrogen bond; **O:** main receptor atom of the main chain; **N:** main donator atom of the main chain; **OE1/OE2**: receptor atom of the side chain; **OD1**: negatively charged atom of the side chain; **OG/OG1**: atom that mainly acts as a donor, but when in contact with another donor may act as a receptor; **NH1/NH2/NZ**: positively charged atom of the side chain from Lys/Arg; **ND2/NE**: donor atom of the side chain; **GlcNAc:** N-acetylglucosamine.
